# The importance of translational research in the study of ocular toxoplasmosis: insights from the 17th International Congress on Toxoplasmosis 2024

**DOI:** 10.1093/femsmc/xtaf003

**Published:** 2025-03-03

**Authors:** Alejandra de-la-Torre, Germán Mejía-Salgado, Gereon Schares

**Affiliations:** Neuroscience Research Group (NEUROS), Neurovitae Center for Neuroscience, Institute of Translational Medicine (IMT), Escuela de Medicina y Ciencias de la Salud, Universidad del Rosario, Bogotá 110321, Colombia; Ophthalmology Interest Group Universidad del Rosario (OIG UR), Escuela de Medicina y Ciencias de la Salud, Universidad del Rosario, Bogota 110321, Colombia; Neuroscience Research Group (NEUROS), Neurovitae Center for Neuroscience, Institute of Translational Medicine (IMT), Escuela de Medicina y Ciencias de la Salud, Universidad del Rosario, Bogotá 110321, Colombia; Ophthalmology Interest Group Universidad del Rosario (OIG UR), Escuela de Medicina y Ciencias de la Salud, Universidad del Rosario, Bogota 110321, Colombia; Health Sciences Faculty, Universidad Autónoma de Bucaramanga UNAB, Bucaramanga 680003, Colombia; Friedrich-Loeffler-Institut, Federal Research Institute for Animal Health, Institute of Epidemiology, National Reference Laboratory for Toxoplasmosis, 17493 Greifswald-Insel Riems, Germany

**Keywords:** *Toxoplasma gondii*, virulence, population structure, clinical research, ocular immune privilege, recurrent toxoplasmosis

## Abstract

Toxoplasmosis is a parasitic infection with significant implications for human health, particularly in its ocular form, which can lead to severe visual impairment. While both basic and clinical research have made considerable strides in understanding the biology and treatment of this parasite, challenges remain. Recent advancements in diagnostics, mainly through multimodal imaging, have improved the identification of active disease and predicting outcomes. Experimental therapies are also emerging, offering new hope for more effective treatments. However, the most critical insight from recent research, particularly emphasized at the 17th International Congress on Toxoplasmosis, is the necessity of a collaborative approach. Integrating basic and clinical research is essential for translating molecular and pathophysiological findings into effective clinical practices. This synergy is crucial for advancing treatment strategies and improving patient outcomes in ocular toxoplasmosis.

## Teaser

Toxoplasmosis is a parasitic infection with significant impacts on human health, particularly in its ocular form and advances in the diagnosis and treatment of ocular toxoplasmosis are urgently needed.

## Introduction

Toxoplasmosis is a parasitic infection affecting humans and other animal vertebrate species. It is estimated that one-third of the world's human population is chronically infected, with up to 80% of these individuals remaining asymptomatic (Park and Nam 2013). Infection occurs congenitally or through ingesting undercooked, infected meat, contaminated vegetables, or water (Goh et al. 2023). Ocular involvement is a notable consequence of *Toxoplasma gondii* infection in 2%–18% of cases, resulting in irreversible retinochoroidal scars, often recurrent episodes, and complications that can threaten vision (Jones and Holland 2010). These complications include vitreous opacities, epiretinal membranes, choroidal neovascularization, and retinal detachment, among others (Park and Nam 2013, Goh et al. 2023). Notably, *T. gondii* is recognized as the leading cause of posterior uveitis worldwide. Since its discovery in tissue cysts in 1908 within the gundi (a hamster-like rodent), extensive research has positioned *T. gondii* among the most thoroughly studied parasites (Dubey 2008).

### Advances in understanding *T. gondii* population structure and virulence factors as base for new diagnostic and therapeutic approaches in ocular toxoplasmosis

The biological study of *T. gondii* has progressed significantly, starting from basic morphological knowledge to an advanced understanding of its life cycle and genotypic variations. Initial studies focused on the morphology of different stages of the parasite. First identified by Nicolle and Manceaux (1908) in the gundi, tachyzoites are lunate and proliferative forms multiplying by endodyogeny (Goldman et al. 1958). In the retina, tachyzoites produce the active form of ocular toxoplasmosis, “retinochoroiditis,” observed as yellowish creamy lesions (Goh et al. 2023).

In contrast, bradyzoites, the encysted stage in tissues, were later characterized by their resistance to gastric digestion and their crucial role in the parasite's life cycle, allowing hosts to become infected through ingesting infected meat (Dubey and Frenkel 1976). In the retina, bradyzoites remain encysted in the retinal cells upon active lesion resolution. They can be the source of further reactivation when they change to their active form, the tachyzoite (Goh et al. 2023). These findings established that retinal cysts are integral to the life cycle of *T. gondii*, independent of host immunity (Ferguson and Hutchison 1987, Dubey et al. 1998).

Over the past decades, advancements have led to the identification of different *T. gondii* genotypes and partially also their associated infection profiles. In Northern America and Europe, the parasite classically comprises three major clonal lineages based on virulence in mouse experimental models: type I, II, III, and atypical (Howe and Sibley 1995, Fernández-Escobar et al. 2022). Type I strains are highly virulent and often associated with laboratory mouse death. In contrast, type II strains are less virulent and represent the most common cause of human infections in these regions. Type III strains were regarded as the least virulent, frequently found in domesticated and wild animals but less commonly in humans (Howe and Sibley 1995, Grigg et al. 2001, Fernández-Escobar et al. 2022). However, more recent studies suggested only marginal differences in the prevalence of particular genotypes in various compartments (i.e. humans, domestic animals, wild animals, and environment) for Europe (Fernández-Escobar et al. 2022). Moreover, previous and more recent studies have observed exceptions from the initial virulence characteristics of clonal types, especially for type III strains (Khan et al. 2009, Taniguchi et al. 2018, Fernández-Escobar et al. 2022).

The worldwide *T. gondii* population structure is very complex, and the phylogenetic analysis of several hundreds of typed isolates worldwide revealed 15 well-defined haplogroups (Su et al. 2012). More recent whole genome sequencing analyses identified 16 haplogroups assorted into six major clades (Lorenzi et al. 2016). The highest genetic diversity in *T. gondii* is observed in South America, suggesting that *T. gondii* may have evolved on this continent and has spread worldwide (Bertranpetit et al. 2017). Several evolutionary bottlenecks, including the predominance of domestic cats as the definitive host (Galal et al. 2022) and commensal rodent species as intermediate hosts (Shwab et al. 2018, Galal et al. 2022), most likely led to the clonal population structure observed in Europe and North America.

Differences in the expression of various virulence factors, which are at least partially the effect of the above-mentioned genotypic differences, may result in differences in the severity of disease manifestations in mice and probably also humans and other intermediate hosts (Sanchez and Besteiro 2021). It was hypothesized that the genotype of an infecting *T. gondii* strain is one of the factors influencing the disease outcome, including ocular toxoplasmosis (Xiao and Yolken 2015). This strain hypothesis is one of the possible explanations for why *T. gondii* is the most frequent cause of posterior uveitis in Latin America because of the highly diverse population structure of the parasite in this region. However, direct typing of *T. gondii* in the case of ocular toxoplasmosis appears challenging due to the usually very limited sample volume of aqueous or vitreous humor and the low *T. gondii* DNA concentrations in these analytes (Fekkar et al. 2008, 2011).

Further research is necessary to improve the direct typing of *T. gondii* in cases of ocular toxoplasmosis to better understand the parasite genotype's effect on the severity of ocular toxoplasmosis. Recent advances to standardize *T. gondii* genotyping represent important prerequisites for reliable direct typing (Joeres et al. 2023, 2024). Indirect typing of *T. gondii* causing ocular infection, i.e. serotyping, appears to be an alternative tool (Mantilla-Muriel et al. 2020), and recent attempts to improve this methodology presented at the 17th International Congress on Toxoplasmosis 2024 appear promising if a translation to human toxoplasmosis is possible (Arranz-Solís et al. 2024).

The rhoptry proteins ROP16 and ROP18, secreted by the parasite upon infection, seem to play pivotal roles in modulating the host immune response. ROP16 phosphorylates host STAT3 and STAT6 transcription factors, altering cytokine profiles and repressing interleukin (IL) 12 signaling necessary for interferon-gamma (IFN-γ) production, which is crucial for the host's survival during infection (Yamamoto et al. 2009, Ong et al. 2010, Hernández-de-los-Ríos et al. 2019). Conversely, ROP18 interferes with host immunity-related GTPases, preventing their interaction with the parasitophorous vacuole membrane, thus aiding the parasite in evading the host's immune system (Steinfeldt et al. 2010). These virulence factors significantly impact the clinical outcomes of *T. gondii* infections, particularly regarding cytokine production and the host's ability to control the infection, as shown in rodent models (Rochet et al. 2019). Which role the rhoptry proteins ROP18 and ROP16 play in ocular toxoplasmosis is not entirely clear. In a cohort of Colombian patients with ocular toxoplasmosis, a higher inflammatory reaction on the eye was associated with the lack of the upstream region of *ROP18* which is in line with a mouse virulent *ROP18* allele (Sánchez et al. 2014). However, in peripheral blood mononuclear cells from individuals with chronic infection (predominantly ocular toxoplasmosis patients), the production of proinflammatory cytokines such as IFN-γ and IL-1b did not seem to be influenced by ROP16 or ROP18 proteins, but by the host's polymorphisms in cytokine genes (Hernández-de-los-Ríos et al. 2019). This indicates that the genetic susceptibility of humans as intermediate hosts may play an important role for the development of ocular toxoplasmosis (Fernández et al. 2016).

In addition to ROP16 and ROP18, *T. gondii* contains and secretes many more rhoptry proteins which are most likely essential in mediating invasion of host cells and host-pathogen interactions (Fernández et al. 2016), as also illustrated by the results of a BioID approach presented at the 17th International Congress on Toxoplasmosis by Stevens et al. (Seeber et al. 2024a, 2024b). Other approaches like CRISPR screens identified further genes essential for invasion, replication, egress, host-parasite interactions, and virulence of *T. gondii* (Butterworth et al. 2023, Tachibana et al. 2023, 2024, Tachibana and Yamamoto 2025), notably further rhoptry but also dense granule (GRA) and microneme (MIC) genes as illustrated by presentations given by Torelli et al. and Tachibana et al. at the 17th International Congress on Toxoplasmosis, respectively (Seeber et al. 2024a, 2024b). Despite these advances, the specific roles of these newly identified proteins and those previously characterized in the pathogenesis of ocular toxoplasmosis remain largely unexplored. Addressing these questions in future translational studies is crucial for advancing our understanding of the disease.

A deeper understanding of *T. gondii* infection has advanced therapeutic approaches beyond the conventional regimen of pyrimethamine and sulfonamides with or without systemic glucocorticoids (Goh et al. 2023). Experimental therapies, such as exosomes and TgMyoA-targeted treatments, show promise. Exosomes derived from mesenchymal stem cells have demonstrated potential in reducing leukocyte infiltration and alleviating uveitis by modulating Th1/Th17 immune responses in *T. gondii*-induced uveitis (Sauer et al. 2012, Bai et al. 2017). Additionally, targeting the MyoA motility complex of *T. gondii*, crucial for parasite movement and invasion, has shown potential, with compounds like KNX-002 inhibiting TgMyoA ATPase activity (Trivedi et al. 2020). While still under investigation, these advanced therapies offer promising avenues for treating ocular toxoplasmosis.

### Clinical research advances

**Figure 1.**
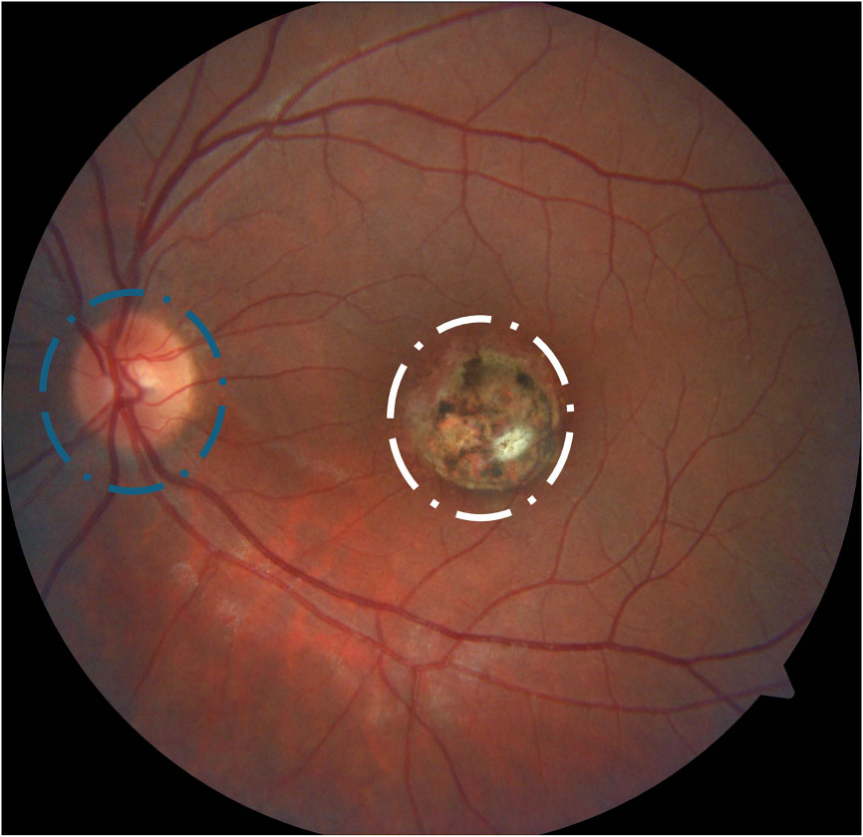
Macular retinochoroidal scar. Fundus photography shows the retina, the optic disc (circle at the left side), and an extensive retinochoroidal scar (circle in the centre) secondary to *Toxoplasma gondii* infection in the macula, the area responsible for detailed and color vision.

Clinical studies have complemented molecular research by providing crucial epidemiological diagnostics and therapeutic insights. A higher prevalence of *T. gondii* infection, recurrence, and more extensive lesions with greater vision impairment due to macular involvement (Fig. 1) have been observed in South American patients (Cifuentes-González et al. 2023a, Karami et al. 2024). This is attributed to the higher prevalence of type I and atypical strains in the Southern Hemisphere compared to Northern countries (Pfaff et al. 2014). Additionally, studies have identified key infection sources specific to different populations. In Colombia, water has been identified as the most contaminated source (Triviño-Valencia et al. 2016), whereas pork is the primary-contaminated meat (Medina Hernández et al. 2022). In contrast, in Australia, lamb is the most contaminated meat (Dawson et al. 2020). Differentiating infection sources for each population is essential for prioritizing governmental measures, such as improving water supply systems, enforcing stricter food safety regulations, and implementing rigorous screening programs for at-risk populations, including pregnant women, who should ideally undergo monthly serological testing (Gómez-Marín et al. 2007).

For diagnosis, the application of multimodal imaging techniques such as color fundus photography, fundus autofluorescence (AF), fluorescein angiography (FFA), and spectral-domain optical coherence tomography (SD-OCT) has advanced the ability to assess and monitor ocular toxoplasmosis (Brandão-de-Resende et al. 2020). In SD-OCT, signs like hyperreflective vitreous dots (80%), thickened (65%) and hyperreflective (61%) retina, choroidal thickening (55%) and hyporeflectivity (61%), and deposits (36%), and posterior hyaloid thickening (35%) indicate active disease and the consequent need for treatment (Oliver et al. 2022). In addition, some imaging findings could predict visual prognosis. For instance, retinal hyporeflective spaces, indicative of liquefactive necrosis, were significantly associated with poorer visual acuity outcomes (20/200 or worse) upon lesion resolution​ (Oliver et al. 2022).

For treatment, clinical research has enabled us to identify at least 24 treatment protocols with no statistically significant differences in outcomes. This diversity in treatment strategies (systemic or intravitreal) has allowed clinicians to tailor therapy based on specific patient factors, allergies to sulfa drugs, adverse reactions, and whether the infection is bilateral or unilateral, among others (Feliciano-Alfonso et al. 2021). A significant advancement in recent decades has been the discovery of the efficacy of prophylactic therapy, which has shown a reduction in recurrence rates by 83% during the first year and 87% during the second year compared to placebo (Cifuentes-González et al. 2023a). Additionally, economic analyses have identified trimethoprim-sulfamethoxazole as the most cost-effective therapy for managing ocular toxoplasmosis. Notably, there is still more to investigate (Álvarez-García et al. 2021). At the 17th International Congress on Toxoplasmosis, Braun et al. proposed the integration of phenotypic screening with forward genetic approaches as a promising and powerful workflow in the search for effective antiparasitic drugs (Seeber et al. 2024b). In addition, further potential drug targets were reported, e.g. by using genomic approaches to identify Toxoplasma-specific targets (Seeber et al. 2024a). Knowledge of putative drugs or drug targets may lead to the development of novel chemotherapeutic approaches for managing ocular toxoplasmosis.

### Summary of key findings on ocular toxoplasmosis reported at the 17th International Congress on Toxoplasmosis

Recent studies presented at the 17th International Congress on Toxoplasmosis provided significant insights into the pathophysiology and clinical management of ocular toxoplasmosis, particularly emphasizing novel mechanisms and clinical correlations.

Taghavi Eraghi et al. highlighted the role of immunosenescence and inflammaging in ocular toxoplasmosis. A retrospective analysis of 290 patients revealed that older age was associated with poorer baseline visual acuity, larger retinal lesions, and increased inflammatory activity (Eraghi et al. 2024). These findings underline the declining local humoral immune response (antibody index) with age, aligning with age-related immune modulation concepts. Moreover, in 24.3% of cases, macular involvement was linked to severe complications such as retinal detachment and poor visual outcomes (Eraghi et al. 2024). This study emphasizes the need to consider age and lesion location in diagnosing and managing ocular toxoplasmosis (Eraghi et al. 2024).

Geiller et al. demonstrated the pivotal role of PD-L1 in maintaining ocular immune privilege. *In vitro* and murine models showed that *T. gondii* infection alters PD-L1 expression in retinal epithelial cells, potentially modulating T-cell activity and inflammation. These findings suggest a dynamic interplay between the parasite and host immune checkpoints, providing a basis for targeted immunomodulatory therapies in ocular toxoplasmosis (Seeber et al. 2024a, 2024b).

Donizete da Silva et al. identified miR-511–5p as a potential biomarker for ocular toxoplasmosis. Elevated levels of this microRNA were associated with higher intraocular pressure in patients with active ocular toxoplasmosis lesions, suggesting its involvement in the pathogenesis of ocular toxoplasmosis. These findings could pave the way for novel prognostic tools and therapeutic strategies targeting microRNA pathways (Seeber et al. 2024a, 2024b).

Lijeskić et al. reported cases of recurrent ocular toxoplasmosis following SARS-CoV-2 infection, suggesting that the altered cytokine milieu during COVID-19 may trigger reactivation of *T. gondii* cysts. Their findings stress the importance of ophthalmologic evaluation in patients with a history of ocular toxoplasmosis and recent SARS-CoV-2 exposure, irrespective of COVID-19 symptomatology (Seeber et al. 2024a, 2024b).

One of the plenary lectures at the congress, delivered by de-la-Torre, focused on the topic “Ocular Toxoplasmosis: Myths and Realities.” De-la-Torre emphasized the significance of varying clinical presentations of ocular toxoplasmosis based on geographic origin. She highlighted that these differences are likely linked to the virulence of specific *T. gondii* strains (de-la-Torre et al. 2013) and genetic polymorphisms in the host (Naranjo-Galvis et al. 2018, 2023), which elicit distinct intraocular and peripheral immune responses. Notably, parasite and host factors cannot be considered separately since they are interdependent (Kalogeropoulos et al. 2022).

The lecture also underscored the importance of prophylactic treatment in preventing recurrences of toxoplasmic retinochoroiditis (Silveira et al. 2002; Cifuentes-González et al. 2023b) and using intravitreal antibiotics as adjuvant therapy for refractory cases with vision-threatening central lesions (Feliciano-Alfonso et al. 2019). Furthermore, de-la-Torre detailed the critical role of advanced imaging modalities, such as optical coherence tomography (OCT), OCT angiography (OCT-A), FFA, and AF, in the diagnostic workup of atypical cases of ocular toxoplasmosis (Invernizzi et al. 2018, Brandão-de-Resende et al. 2020).

## Conclusion

Although significant knowledge has been gained since the discovery of *T. gondii*, much remains to be done. Future research should address the disruption of IFN-γ-signaling by South American strains and explore the possibility of therapies, e.g. to boost IFN-γ production, such as CD4+ memory T-cell stimulation. Developing immune-based interventions and chemotherapeutics capable of penetrating tissue cysts in intraocular tissues is critical, alongside identifying drugs specifically designed to eliminate these cysts. Additionally, personalized medicine approaches should evaluate differential therapeutic responses based on host susceptibility and the genetic diversity of *T. gondii* strains. These strategies require integrated basic and clinical research to advance effective treatments for ocular toxoplasmosis, as neither group can accomplish this alone. Strengthening translational research is essential, so clinical findings can inform basic research, and insights into molecular and pathophysiological pathways can become relevant for clinicians.

This was one of the central themes of the 17th International Congress on Toxoplasmosis 2024 (Seeber et al. 2024a, 2024b), where specific examples highlighted the necessity of integrative efforts. For instance, geographic variations in clinical presentations of ocular toxoplasmosis linked to strain virulence and host genetic polymorphisms were emphasized. The importance of prophylactic treatment and advanced imaging modalities for diagnosis were underscored. Additionally, studies on age-related immune changes highlighted the impact of immunosenescence and inflammaging on disease severity. An altered cytokine milieu as the underlying cause for the reactivation of *T. gondii* cysts causing recurrent ocular toxoplasmosis following SARS-CoV-2 infection may also apply for other virus infections and needs further investigation. The identification of microRNA 511–5p as a potential biomarker for disease activity in ocular toxoplasmosis may trigger research on further microRNAs as useful diagnostic or prognostic markers. The identification of new drug targets and the establishment of novel workflow in the search for effective antiparasitic drugs may lead to the identification of novel chemotherapeutics, potentially suitable for treatment in ocular toxoplasmosis. These examples illustrate how multidisciplinary research and clinical collaborations are essential to advancing our understanding and improving patient outcomes in ocular toxoplasmosis.
